# Application of High-Pressure Processing to Assure the Storage Stability of Unfiltered Lager Beer

**DOI:** 10.3390/molecules25102414

**Published:** 2020-05-21

**Authors:** Kateřina Štulíková, Tomáš Bulíř, Jakub Nešpor, Lukáš Jelínek, Marcel Karabín, Pavel Dostálek

**Affiliations:** Department of Biotechnology, Faculty of Food and Biochemical Technology, University of Chemistry and Technology, Technicka 5, Prague 6-Dejvice, 166 28 Prague, Czech Republic; bulirt@vscht.cz (T.B.); nesporj@vscht.cz (J.N.); lukas.jelinek@vscht.cz (L.J.); marcel.karabin@vscht.cz (M.K.); pavel.dostalek@vscht.cz (P.D.)

**Keywords:** unfiltered beer, high-pressure processing, pascalization, shelf-life

## Abstract

Due to the increasing popularity of unfiltered beer, new methods for its preservation are needed. High-pressure processing (HPP) was applied as a final treatment of packed beer in order to assure storage stability and to retain the desired product quality. Pressures of 250 MPa and 550 MPa for 5 min were used to process unfiltered lager beers. The impact of pressure on basic analytical characteristics was evaluated, and foam stability, the content of carbonyl compounds and sensory properties were monitored during two months of storage. Most of the basic analytical parameters remained unaffected after pressure treatment, and a beneficial effect on foam stability was demonstrated. Changes in the concentration of staling aldehydes were observed during storage. Some features of the sensory profile were affected by HPP as well as by the time of storage. Our study evaluated the suitability of HPP as a novel method for shelf-life extension of unfiltered lager beer.

## 1. Introduction

Unfiltered beer is characterized by the presence of yeast in the final product. Filtration as a processing step assuring the removal of yeast before packaging is omitted for various reasons, mainly in order to preserve the natural beer character, manifested by fullness in taste and distinct flavor [1,2]. Unfiltered beer is also presumed to be a richer source of B vitamins compared to the filtered product [3]. Further health benefits are connected to beta-glucans present in the yeast cell wall, which have immuno-stimulating and prebiotic effects [4,5]. The production of unfiltered beer has increased due to the rapid growth of craft breweries, whose production typically aims to eliminate additional industrial processing steps in order to preserve the authentic character of their product [6,7]. Consequently, in response, industrial breweries also started producing unfiltered beers so as to expand the diversity of their portfolios and attract a wider range of customers [8]. 

Regardless of the production capacity of the brewery, storage stability of beer is a crucial issue for any brewer [9]. The conventional approach to maintain a stable quality of product for the duration of its desired shelf-life period is thermal pasteurization [10]. Although highly reliable, in assuring microbiological stability, pasteurization is also associated with some detrimental effects on beer sensorial and colloidal properties. Thermal treatment during pasteurization is known to accelerate the beer aging process, including an increase in color, decrease in bitterness, development of stale flavor and other quality deteriorations [11,12]. However, if pasteurization is omitted and the fresh beer is kept refrigerated until consumed a significantly shorter shelf-life, within the range of several days or weeks, is assured [13]. Therefore, this approach is mostly pursued by breweries with production destined for local customers [14]. 

High-pressure processing (HPP), also called pascalization, is a non-thermal stabilizing method that has expanded throughout the food and beverage industry. The main benefits of HPP are preservation of freshness and appearance, nutritional values and vitamin content, and therefore it is widely applied, especially for fruit juices and vegetable products [15,16]. From a legal point of view, in food products where HPP is applied, market authorization might be required based on the Novel Foods Regulation of the European Union. However, in practice, the approach differs in individual member states. Generally, HPP is no longer considered as a novel method, but despite that, additional safety assessments of pressurized products might be requisited [17]. Other types of applications have been investigated, including the potential use as an alternative for the preservation of unfiltered beer [18]. The efficiency of HPP in microbiological stabilization of beer is comparable to heat pasteurization and according to results of experiments so far conducted, no impact on the essential beer quality parameters (ethanol content, pH, extract, bitterness) has been observed [19,20,21]. Applications of HPP in other stages of the brewing process have also been experimentally tested and some beneficial effects have been proven, especially during the mashing step, where pressure enhances starch saccharification [18]. 

Microbiological stabilization by HPP is assured by the effect of denaturation of nucleic acids and proteins, causing irreversible changes in yeast and bacterial cell morphology and subsequent lethality [22]. However, because the permeability of the cell membrane changes, the intracellular mass leaks out [23,24]. This could have an impact on some crucial quality parameters of unfiltered beer and its stability during storage, but it has not yet been sufficiently investigated. 

One of these attributes for a lager beer style is its thick creamy head. Beer foam is a result of mutual interactions of foam-positive and foam-negative compounds. Foam-positive constituents are represented by structurally varied groups of proteins, hop acids, polyphenols and non-starch polysaccharides, whereas the foam-negative components are mainly lipids [25,26]. Foam stability is also related to the activity of proteinase A (pro-A, EC 3.4.23.25), an intracellular yeast protease that can penetrate to the extracellular environment (e.g., in stress conditions) and cause cleavage of foam-active proteins, thereby reducing the foaming potential [27].

A suitable method for stabilizing unfiltered beer should preserve its fresh taste and drinkability, attributes that are typically appreciated for Pilsner lagers. The freshness of beer can be defined by the absence of staling flavors, which are mostly caused by carbonyl compounds originating in a plethora of oxidative reactions taking place in beer during the production process and packaging [28,29]. Conditions after packaging, mainly storage temperature, are also important factors related to the development of stale flavor marks in beer [30]. The influence of HPP on the concentration of carbonyl compounds associated with beer aging is not yet known and neither is the overall impact on the sensory properties of unfiltered beer. Hence, the aim of our study was to verify whether HPP causes any changes in the basic parameters of unfiltered lager beer (extract, alcohol content, pH, color) and to describe its impact on foam stability and the content of carbonyl compounds associated with beer staling. Additionally, sensorial stability and the role of storage temperature on quality changes were evaluated.

## 2. Results and Discussion

### 2.1. Impact of High-Pressure Processing on the Basic Analytical Parameters of Unfiltered Beer

To assess the applicability of HPP for extension of shelf-life of unfiltered beer we tested two processing pressures (250 MPa and 550 MPa) for 5 min at 25 °C. Basic analytical parameters—original extract, alcohol content, apparent extract, pH and color—were measured immediately after processing. These parameters served for routine quality checks of the final packaged beer and some of them for defining certain beer types and legislative categorization [31]. Therefore, it was desirable that the final processing method did not cause any deviations and the values of these parameters remained unaltered. The results confirmed that HPP did not cause changes in original and apparent extracts, pH, alcohol content and density. However, beer color increased significantly in the sample processed at 550 MPa (Table 1). These results are in agreement with Buzrul et al. [19], who showed that an increase in color correlated with higher pressure and longer processing time. In contrast, other experimental data showed that beer color was not influenced by pressure processing [21,32,33]. Changes in color upon processing were mainly a result of non-enzymatic browning (Maillard reactions), induced by pressures above 400 MPa at a temperature of 60 °C [34]. Beer color can also increase due to the oxidation of polyphenols [35]. 

### 2.2. Impact of High-Pressure Processing on the Foam Stability of Unfiltered Beer

**Figure 1:**
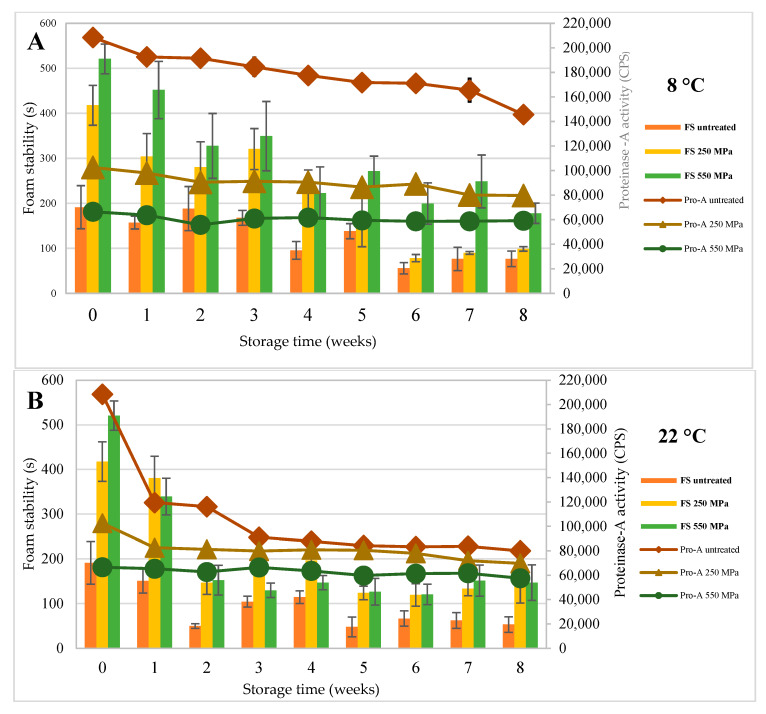
Impact of high-pressure treatment at 250 MPa for 5 min or 550 MPa for 5 min on the foam stability (FS, in columns) and the activity of proteinase A (pro-A, inline connections) compared with the untreated sample during 8 weeks of storage at (**A**) 8 °C or (**B**) 22 °C.

Beer foam stability (FS) is an important quality parameter due to its direct link to the customer’s impression of beverage quality, which is being formed even before the start of drinking. Our results showed that the FS of unfiltered beer increased with HPP and higher processing pressure was associated with the highest FS immediately after treatment and after two months of storage (Figure 1). These results are in accordance with Pérez-Lamela et al. [33].

We evaluated the impact of temperature on changes in FS during storage. Over the two-month monitoring period, FS declined significantly in all samples including the control samples and final FS values of the sample sets stored at room temperature were comparable to those that were kept refrigerated at 8 °C. However, the decrease in FS was more rapid during the first three weeks of storage in beers kept at room temperature, while in the refrigerated samples, the decline was linear over the whole storage period. The decline of FS could be related to the release of fatty acids from the yeast cells as a result of autolysis [36]. Fatty acids gather on the liquid–gas interphase, where they interfere with foam-stabilizing proteins and cause coalescence of air bubbles, leading to foam destruction [25]. Higher storage temperature accelerates yeast autolysis, which explains the faster decline of FS in samples kept at room temperature [36]. 

The highest pro-A activity was found in the control sample at the beginning of storage. In the 550 MPa-processed beer, enzyme activity remained the lowest for the whole storage period, whereas in the 250 MPa-treated and in the control samples, pro-A activity further declined during storage (Figure 1). These findings indicate that pro-A is inactivated by a pressure of 550 MPa. In samples stored at 22 °C, the decrease of pro-A activity in control beer could be associated with the release of yeast inhibitors, such as IA_3_, which binds to the enzyme and causes a formation of an inactive complex IA_3_, pro-A [37].

### 2.3. Changes in Concentrations of Carbonyl Compounds after Processing and during Storage

Flavor instability is related to changes in concentrations of many different compounds, however, carbonyl compounds have been identified as markers of beer staleness [38]. We analyzed the aldehyde content of beer after pascalization to examine the effect of different pressures (250 MPa and 550 MPa). In addition, pascalized beers were stored at different temperatures to assess the impact of higher temperatures on reactions connected to beer aging. Obtained data were processed by the Shapiro–Wilk test which has shown data were normally distributed (data not shown). 

Table 2 shows that concentrations of compounds from a group of Strecker aldehydes (SA, 2-methylpropanal, 2-methylbutanal, 3-methylbutanal and benzaldehyde) increased proportionally with the applied pressure. The increase in SA in pressurized beers was even higher in samples stored at 22 °C and the highest concentration of SA was found in the sample treated with 550 MPa at the end of the 2-month storage period at 22 °C. SA are degradation products of amino acids, or they can result from oxidative degradation of isohumulones from hops. In fresh beer, the majority of aldehydes is present in the form of adducts with amino acids, proteins or sulfites. During storage, they are released from the bound-state and contribute to the stale flavor [39,40]. When their concentration in beer exceeds the sensory threshold, undesired attributes such as solvent, malty, cherry or almond occur. The results indicate that HPP could enhance the release of SA from adduct forms or cause their formation de novo. Further examination is necessary to clarify the mechanism of SA formation after HPP treatment and during storage. 

Another group of staling compounds consists of linear aldehydes (hexanal, heptanal, octanal), which are degradation products of fatty acids (linoleic and oleic) mainly originating from malt. Their content in beer after processing increased linearly with pressure and after two months of storage at 22 °C, the content increased significantly in all samples, including the control (Table 2). These results imply that besides precursors from malt, another source of linear aldehydes is fatty acids released from yeasts as a result of autolysis during storage [41]. 

The formation of staling compounds in beer is caused by a plethora of oxidative reactions occurring in beer. Some of them are directly accelerated by the presence of oxygen [35]. Regarding the plastic bottles used as a final packaging material for pressurized beers, the uptake of oxygen through the material could not be fully prevented, which was another cause of the increase of the carbonyl concentrations in beers during storage. 

In untreated beers (controls), concentrations of the majority of carbonyls were lower in beers stored at higher temperatures compared to refrigerated samples. Similarly, in beers processed at 250 MPa kept at 22 °C, the concentration of some carbonyls after two months was lower than after one month of storage. The decrease was related to the activity of yeast reducing enzymes capable of carbonyl reduction to the respective alcohols [40]. These reactions were inhibited in cold storage temperatures. At lower processing pressures, changes in enzyme conformations can be reversible, and therefore the reducing complexes in beer processed at 250 MPa were still active [42]. 

**Figure 2:**
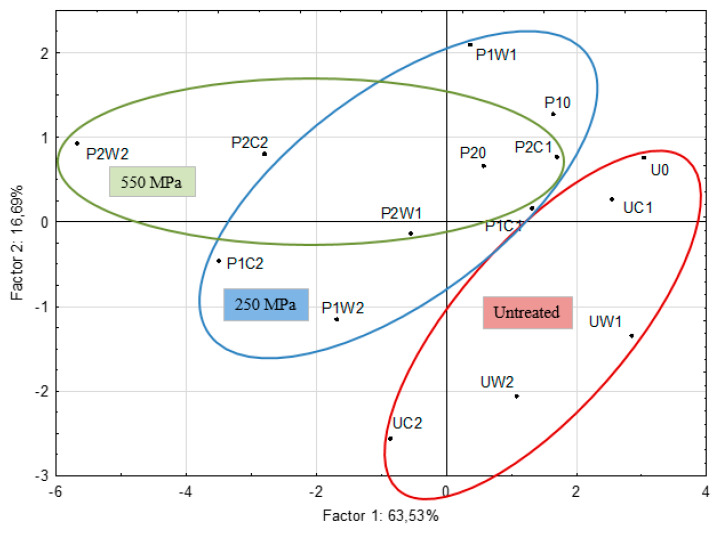
Principal component analysis according to the content of staling aldehydes in beers processed at different pressures (250 MPa or 550 MPa) and stored in cold (8 °C) or at room temperature (22 °C) for 1 or 2 months. Control sample was beer untreated by pressure processing. Particular samples are coded according to the type of treatment as listed in Table 2. Samples are clustered in 3 groups according to the type of processing (untreated, processed at 250 MPa or at 550 MPa).

For clearer visualization of differences between samples principal component analysis (PCA) of data obtained was performed. PCA identified two factors that explained 80.21% of the variation in carbonyl concentrations (Figure 2). Contribution to both factors given by the individual variables is summarized in Table 3. The percentage of total variance for each factor is depicted in Figure 2. Samples within the PCA scatter plot were clustered into three groups with respect to the pressure treatment (untreated, 250 MPa and 550 MPa treatment, respectively). Separation of the samples indicates that higher pressure treatment, as well as storage at higher temperatures and the two-month duration of storage were associated with the highest concentrations of staling aldehydes. 

### 2.4. Effect of HPP on the Sensory Properties

**Figure 3:**
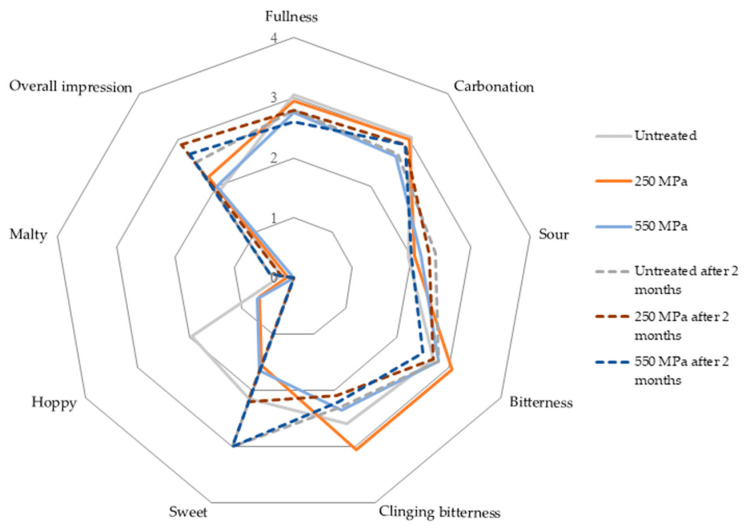
A radar plot of sensory parameters obtained from the descriptive analysis of beers right after processing and after two months of storage at 8 °C. Overall impression was rated on a scale from 1 to 5 (1 = very good, 5 = very poor), other parameters were evaluated on a scale from 0 to 5 (0 = not present, 5 = very strong).

The sensory profile of the Pilsner lager beer type is characterized by medium fullness, balanced bitterness of medium intensity, typical hoppy and malty aromas and slight diacetyl flavor [43]. Figure 3 shows differences in ratings of selected sensory descriptors in evaluated beers, which were statistically relevant to both pressure treatment as well as to storage time, with the level of significance set at *p* < 0.05. Processing at higher pressure caused a decrease in fullness, bitterness, and hoppy flavor, which indicates that a pressure of 550 MPa could accelerate the degradation of polyphenols, hop bitter acids and aromatic compounds. Changes in all pressurized as well as in untreated beers after two months of storage comprised increased intensity of sour, malty and sweet flavors, whereas the intensity of hoppy flavor and clinging bitterness decreased. The increased intensity of malty flavor and sweet taste are related to the increase in carbonyl compounds during storage, as was also confirmed by SPME-GC/MS analysis. The rating of overall impression decreased significantly in all samples after two-month storage but in the worst case, was still rated as good.

## 3. Materials and Methods

### 3.1. Beer Samples and High-Pressure Treatment

Unfiltered Pilsner lager beer (12 °C) was donated from a local industrial-scale brewery. From kegs, the beer was filled into 1 L plastic (PET) bottles with 2% headspace volume left, using a manual bottling machine (Pegas, Vilnius, Lithuania) under a protective atmosphere of CO_2_. After packaging, samples were subjected to HPP treatment. Different processing pressures were selected according to previously published studies, as presentable for the low and high limit for efficient microbial stabilization of beer [18]. Samples were divided into three groups; the first group was subjected to pressure treatment of 250 MPa for 5 min, the second was processed at 550 MPa for 5 min at 25 °C. 

During pressurization, temperature was held at 25 °C. The remainder of samples without processing were used as controls. HPP was performed using Hiperbaric 135 pressurizing equipment (Hiperbaric, Burgos, Spain). Samples were then stored for two months in a refrigerator at 8 °C or in a dark room at 22 °C.

### 3.2. Measurement of Basic Analytical Parameters

Prior to analysis, yeast cells were removed by centrifugation (6000 rpm, 10 °C, 10 min.). Beer was degassed in an ultrasound bath at 10 °C for 10 min. The original extract, apparent extract, alcohol content, pH and color of beer before and after HPP treatment were measured using a DMA 4500 M Beer Alcolyser (Anton Paar, Graz, Austria) according to the principle of MEBAK 2.9.6.3 and MEBAK 2.12.2.

### 3.3. Foam Stability and the Activity of Proteinase A

Foam stability (FS) was measured by a method developed by Košin, Šavel, Brányik and Ulmann (2017), using a foam stability analyzer (1-CUBE, Havlíčkův Brod, Czech Republic). Thirty mL of beer in a 100 mL beaker cleaned with ethanol was used for the analysis and stability was expressed as time until the level of beer foam generated by aeration decreased to a level 5 mm above the beer level. 

The activity of proteinase A was evaluated using a method previously published by Chen et al. [44]. Fluorescent-quenching substrate for proteinase A, MOCAc-Ala-Pro-Ala-Lys-Phe-Phe-Arg-Leu-Lys(Dnp)-NH2, was obtained from the Peptide Institute, Inc. (Osaka, Japan). Twenty-four µL of 0.365 mg/mL solution of the substrate in dimethyl sulfoxide (Sigma-Aldrich, St. Louis, MO, USA) was added to a mixture of 1500 µL of phospho-citrate buffer (pH adjusted to 5.5), 1380 µL of distilled water and 100 µL of beer. After 30 min of incubation at 37 °C, the sample was placed in an 80 °C water bath to arrest the quenching reaction. Fluorescence was measured by Spectrofluorimeter Fluoromax-4P (Horiba Jobin Yvon, Kyoto, Japan) at an excitation wavelength of 328 nm and an emission wavelength of 393 nm. Results were expressed in counts per second (CPS). 

Foam stability and the activity of pro-A were measured at weekly intervals for a duration of 2 months. Both refrigerated samples as well as the samples stored at room temperature were analyzed to determine the impact of temperature on parameter changes. 

### 3.4. Assessment of Selected Volatile Carbonyl Compounds

For the analysis of carbonyl compounds by SPME-GC/MS, 50 mL of the beer was degassed and centrifuged. Ten mL of the sample were then added to a 20 mL dark glass vial containing 2 g NaCl (Penta, Prague, Czech Republic) and 100 µL of 52.6 mg/L 3-fluorobenzaldehyde as an internal standard (Sigma-Aldrich, St. Louis, MO, USA). The derivatization agent for each analysis was prepared in the same way as the samples, containing 2 g of NaCl, 10 mL of demineralized water (Mili-G Millipore, Millipore, Burlington, MA, USA) and 200 µL of a 5978 mg/L solution of *o*-(2,3,4,5,6-pentafluorobenzyl)hydroxylamine hydrochloride (PFBOA, Fluka, Munich, Germany; ≥99% purity). All vials were sealed with a PTFE-silicone septa (Supelco, Bellefonte, PA, USA).

Samples were analyzed by SPME-GC/MS. All parameters and the procedure were in accordance with the method of Andrés-Iglesias et al. [38], except for the type of capillary column, which was an HP-5MS 60 m × 0.25 mm × 0.25 mm (Agilent Technologies, Santa Clara, CA, USA). Compounds were identified by comparison of their mass spectra with standard mass spectra as listed in the NIST MS 2.0 spectral database (National Institute of Standards and Technology, Gaithersburg, MD, USA), and by comparison of retention times with standards. Quantification was performed from normalized peak areas using an internal standard, by three-point external calibration via the addition of different amounts of external standards: 2-methylpropanal (≥99%), 2-methylbutanal (≥95%), 3-methylbutanal (≥97%), (2E)-non-2-enal (≥97%), hexanal (≥98%, Sigma-Aldrich, St. Louis, MO, USA), benzaldehyde (≥98%, Alfa Aesar, Haverhill, MA, USA), heptanal (≥97%), octanal (98%, Merck, Darmstadt, Germany). Samples were analyzed right after HPP processing, and then after 1 and 2 months of storage at 8 °C or 22 °C. 

### 3.5. Sensory Evaluation

To determine the influence of HPP on the taste and flavor, beer was subjected to sensory evaluation. Descriptive analysis was performed according to a method of sensory analysis by the American Society of Brewing Chemists (ASBC Methods of Analysis). The panel comprised 8 trained panelists (age 26–53 years, 4 females and 4 males) from a local industrial-scale brewery. Tasters evaluated the selected quality attributes on an intensity scale from 0 to 5, (0 = not present, 5 = very strong), and the overall impression was marked on a scale from 1 to 5 (1 = very good, 5 = very poor). Attributes included general taste and mouthfeel characters: fullness, carbonation, astringency, sourness, bitterness, clinging bitterness, sweet and hoppy flavors, diacetyl, malty, aged. Beer was tasted right after HPP processing and after two months of storage at 8 °C.

### 3.6. Statistical Analysis

All analyses except for the sensory descriptive analysis were performed in triplicate and results were expressed as means ± standard deviations (SD). The student’s T-test was used to evaluate differences between the results, with statistical significance set at *p* < 0.05. Furthermore, the Shapiro–Wilk test of normality and one-way analysis of variance was performed with the data from GC/MS analysis (data not shown). Principal component analysis (PCA) was conducted to evaluate the influence of HPP and storage at different temperatures on the content of carbonyl compounds. Statistical analyses were performed using Statistica data analysis software, version 13 (TIBCO Software Inc., 2018, Palo Alto, CA, USA).

## 4. Conclusions

The suitability of HPP as a final treatment method for unfiltered lager beer was appraised. HPP did not cause alterations in basic analytical parameters. Furthermore, high-pressure inactivated pro-A, which contributed to better foam stability of the unfiltered beer.

HPP belongs to commercially implemented technologies for microbial stabilization of various food products, and additionally, it retains freshness and nutritional properties. However, an increase in carbonyl compounds associated with beer staling during storage after packaging was not prevented by HPP. More investigations conducted under a variety of storage conditions and pressures are necessary to further determine the impact of HPP on mechanisms of beer aging.

Some changes in selected sensory features that define the lager beer style were related to the intensity of pressure treatment as well as to the storage period. Nevertheless, the overall sensory quality of pressurized beer was still rated as good.

Albeit the use of HPP for industrial production of unfiltered beer would require extensive optimization regarding the intensity of applied pressure and the packaging materials, we believe the innovative character of the method represents a promising potential for future applications in the brewing industry.

## Figures and Tables

**Table 1:** Physico-chemical parameters of beers after high-pressure processing (HPP) at different pressures.

Sample	Untreated	250 MPa	550 MPa
Original gravity (% *w*/*w*)	12.26 ± 0.01	12.18 ± 0.01	12.22 ± 0.01
Alcohol (% vol.)	4.84	4.80	4.81
Apparent extract (% *w*/*w*)	3.19 ± 0.01	3.20 ± 0.01	3.21 ± 0.01
pH	4.73	4.73 ± 0.01	4.73 ± 0.01
Color (EBC)	12.5 ± 0.2	12.3 ± 0.4	14.1 ± 0.1 *
Density (g/cm^3^)	1.0106	1.0107	1.0107

Values expressed as mean ± standard deviation (*n* = 3); * indicates significantly different values (*p* < 0.05).

**Table 2:** Concentrations of staling aldehydes (µg/L) in beers processed at different pressures (250 MPa or 550 MPa) and stored in cold (8 °C) or at room temperature (22 °C) for 1 or 2 months. Samples are coded according to: U/P1/P2 relates to the type of processing—untreated, 250 MPa, 550 MPa, respectively; C/W relates to “cold” (8 °C) or “warm” (22 °C) storage temperature and the last letter 0/1/2 relates to the storage time—0, 1 or 2 months.

Sample Code	Processing Pressure (MPa) ^a^	Storage Temperature (°C)	Length of Storage (Months)	2-Methyl Propanal	2-Methyl Butanal	3-Methyl Butanal	Benzaldehyde	Heptanal	Hexanal	Octanal	(2E)-Non-2-enal
U0		-	0	14 ± 2	8	15	2 ± 1	1	2	3	2
UC1	-	8	1	14 ± 2	8 ± 1	17 ± 1	3 ± 1	1	3	5 ± 2	2 ± 1
UW1	-	22	1	12	6	10	4	1	4	8 ± 1	1 ± 1
UC2	-	8	2	17 ± 2	9 ± 1	25 ± 1	16 ± 6	7	5	6 ± 2	4 ± 1
UW2	-	22	2	11 ± 2	6 ± 1	14 ± 1	13 ± 2	5 ± 1	4 ± 1	5	2
P10	250	-	0	26 ± 2	12 ± 1	23 ± 1	3 ± 1	1	3	4	2
P1C1	250	8	1	23 ± 4	12 ± 1	23 ± 1	8 ± 3	2	3	6 ± 2	2 ± 1
P1W1	250	22	1	43 ± 2	17 ± 2	31 ± 1	5 ± 1	1	5 ± 1	4 ± 1	3 ± 1
P1C2	250	8	2	41 ± 4	19 ± 3	39 ± 2	17	6 ± 1	8 ± 1	5 ± 1	7 ± 2
P1W2	250	22	2	26 ± 11	11 ± 3	23 ± 2	12 ± 3	5 ± 2	7 ± 1	3 ± 1	4 ± 1
P20	550	-	0	30 ± 1	13 ± 1	24 ± 1	6	2	4	5 ± 1	3
P2C1	550	8	1	24 ± 1	10 ± 1	20	6	1	4	4 ± 1	2 ± 1
P2W1	550	22	1	37 ± 1	15	29	15 ± 1	2	4	5 ± 1	3
P2C2	550	8	2	46 ± 4	19 ± 1	39 ± 2	14 ± 1	4 ± 2	7	4	6 ± 1
P2W2	550	22	2	80 ± 10	25 ± 2	48 ± 2	24 ± 7	5 ± 2	8 ± 1	5	8 ± 1

^a^ Control sample was beer untreated by pressure. Results are expressed as mean ± standard deviation (*n* = 3).

**Table 3:** Factor contributions (loadings) of the individual carbonyl compounds.

	Factor 1	Factor 2
**2-Methylpropanal**	−0.835952	0.475608
**2-Methylbutanal**	−0.850818	0.491356
**3-Methylbutanal**	−0.914484	0.352284
**Hexanal**	−0.943668	−0.124385
**Heptanal**	−0.714630	−0.600826
**Octanal**	0.125436	−0.503644
**Benzaldehyde**	−0.904390	−0.315189
**Nonenal**	−0.960025	0.095859
